# Maximum information measurement for qubit states

**DOI:** 10.1038/s41598-024-62446-9

**Published:** 2024-05-24

**Authors:** Árpád Varga, Peter Adam, János A. Bergou

**Affiliations:** 1 Institute of Physics, University of Pécs, Pécs, Ifjúság útja 6, 7624 Hungary; 2 Institute for Solid State Physics and Optics, HUN-REN Wigner Research Centre for Physics, Budapest, P.O. Box 49, 1525 Hungary; 3 Department of Physics and Astronomy, Hunter College and the Graduate Center of the City University of New York, 695 Park Avenue, New York, NY 10065 USA

**Keywords:** Quantum information, Qubits

## Abstract

We determine the optimal measurement that maximizes the average information gain about the state of a qubit system. The qubit is prepared in one of two known states with known prior probabilities. To treat the problem analytically we employ the formalism developed for the maximum confidence quantum state discrimination strategy and obtain the POVM which optimizes the information gain for the entire parameter space of the system. We show that the optimal measurement coincides exactly with the minimum-error quantum measurement only for two pure states, or when the two states have the same Bloch radius or they are on the same diagonal of the Bloch disk.

## Introduction

In quantum information the carriers of information are quantum systems and information is encoded in their states. Extracting this information is a central problem in quantum information processing and it can be done by determining the state via measurements^1^.

In many quantum communication schemes, information is the state itself. In these schemes a sender, Alice, prepares an ensemble of quantum systems, each in a state from a set of *n* known states, $\{\rho _{j}|j=1,\ldots ,n\}$, the letter states. The weight of state $\rho _{j}$ in this initial ensemble is $\eta _j$, called the *a priori* probability or simply prior. Alice then randomly draws a system from this initial ensemble and sends it to the receiver, Bob. The set of possible states as well as their priors are also known to the receiver whose task is to identify the state of the system he received. If the states are mutually orthogonal the task is easy: Bob sets up detectors along these orthogonal directions and a click in one of them will perfectly determine the input. However, if the possible states are not mutually orthogonal, the problem is highly nontrivial. Bob needs to choose a figure of merit and find a measurement which is optimal with this respect. Accordingly, several strategies have been developed with respect to various criteria. Optimization, in general, leads to complex measurement strategies often involving generalized measurements. Some of the frequently employed strategies are discrimination with minimum error (ME)^2–6^, unambiguous discrimination (UD)^7–12^, and maximum confidence (MC)^13–16^ discrimination.

The ME strategy was first introduced in Refs.^2–4^ for two states (pure or mixed) with arbitrary priors. In this strategy, every time Bob receives a system he has to make a guess about its state based on the outcome of his measurement. The price to pay is that errors must be allowed. In the optimal strategy the average probability of error is minimized. The ME strategy involving more than two states is known in some special cases only.

The UD strategy was first introduced in Refs.^7–9^ for two pure states with equal priors and was later generalized for arbitrary prior probabilities in Ref.^10^. In the UD strategy, no errors are allowed. The price to pay is that Bob must be allowed to return inconclusive answers. In the optimal strategy the average probability of inconclusive answers is minimized. An important result states that UD is possible if the states are linearly independent^17^, which is not a requirement for ME. The UD strategy is successively used in sequential state discrimination, which is a strategy for *N* separate receivers^18–22^.

We note that each strategy has its own advantages and drawbacks when we try to apply them for a general measurement problem. It is difficult to find measurements realizing unambiguous discrimination for mixed states, but it is relatively easy to generalize this strategy for more than two states, at least in principle^23^. The ME strategy handles mixed states and pure states on equal footing but is hard to generalize for more than two states, except for some special, highly symmetric cases^24,25^ (although progress has been made recently in this area^26,27^).

Another independent strategy, called Maximum Confidence (MC), was introduced in Ref.^13^. The aim of the MC strategy is to construct a measurement which maximizes the confidence $C_{j}$: the conditional probability that detector *j* clicks provided that the state $\rho _{j}$ was prepared. In the case of linearly independent states this strategy coincides with the UD strategy. However, when the states to be discriminated are not linearly independent, this is an independent strategy^28^. For further developments in this line of state discrimination studies we refer to the recent reviews^14–16^.

In quantum communication one can look for a measurement strategy maximizing the mutual information between the communicating parties. In this problem the sender sends a sequence of individual quantum systems, each taken from a given set of known states ${\mathscr {E}}=\{\rho _{j}|j=1,\ldots ,n\}$, and the receiver measures them one by one, possibly by a POVM with the POVM elements $\Pi _{m}$ where $m=1,\ldots ,N$. Our task is to maximize the Shannon mutual information^2,3,29^,

1 $$\begin{aligned} I\left( m:j\right) = \sum _{m,j} p(m|j) \eta _j \ln \frac{p(m|j)}{p(m)}, \end{aligned}$$

between the measurement outcomes *m* and the input states *j*, where $\eta _j$ is the *a priori* probability of the state *j*, and $p(m|j)=\textrm{Tr}(\Pi _m\rho _j)$ is the conditional probability of getting the measurement result *m*, given that the state $\rho _j$ was prepared.

For a given set of states and their *a priori* probabilities, the problem is to find a measurement which maximizes the mutual information. While the mutual information is known to obey the Holevo bound^30^, it is important to determine the accessible information, which is the actual maximum over all possible measurements. This is a special problem in state discrimination: we want to maximize the correlation between state preparation and measurement outcomes, i.e., we want to devise a measurement strategy that yields maximum information about which state was prepared. We will refer to such a measurement as maximum information (MI) measurement. Solution to this problem is known only for a few special cases. Even determining the amount of information which can be encoded in a given quantum system is a nontrivial task^31–36^. We note that the problem we address consists in maximizing mutual information between classical random variables linked via a quantum encoding–decoding scheme. Therefore, it is related to the calculation of channel capacities. It is, however, different from that of quantum channel capacity concepts maximizing quantum (as opposed to classical) mutual information^37–41^.

The number of POVM elements needed to maximize the information gain is in general unknown. It is known^42^ that for any ensemble in *d* dimensions there is an optimal strategy with at most *N* elements, where $d\le N \le d^2$. Sasaki et al.^43^ showed that for the case of real states (that is, states with a real density operator), the upper bound is $d(d+1)/2$. They also gave explicit solutions for the case of real and symmetric states in two dimensions, and showed that at most three POVM elements are necessary. Levitin^44^ conjectured that if the number of the possible states is $N \le d$, the optimal measurement will always be a von Neumann measurement. This conjecture was proven to hold for two pure states in arbitrary dimensions by Levitin. However, Shor^45^ gave a counterexample involving three real pure states in three dimensions (qutrits). Considering qubits only, Keil^46^ proved that von Neumann measurements are always optimal for two general states.

Fuchs and Peres^47^ studied numerically the trade-off between the information gain and the measurement induced disturbance. Ban et al.^48^ gave analytic results for pure binary signal states, and showed the connection between the ME measurement and the MI measurement for this special case. Řeháček et al.^49^ gave an iterative algorithm to find the optimal POVM for the accessible information and illustrated the method on an example in three dimensions. There are also lower^50^ and upper^51,52^ bounds to the accessible information for simple cases, which depend explicitly only on the message ensemble.

In this paper we consider the problem of finding the optimal measurement to maximize the mutual information for a general qubit system. Our approach makes use of the method developed for the maximum confidence strategy and leads to analytical insight. In particular, we determine the POVM in parametric form with a single parameter, which maximizes the information gain for the entire parameter space of the system.

## Results

### Information gain and confidence probabilities: General formalism

We begin with a study of the simplest case of two qubit states in a two-dimensional Hilbert space and present an alternative derivation of Eq. (1) for this case. We cast the result to a form that shows the intrinsic connection of information gain with the confidence probabilities, introduced in Ref.^13^.

Recall that we consider a two-party protocol with a sender, Alice, and a receiver, Bob. Alice prepares an ensemble of qubit systems where each qubit is either in the state $\rho _1$ or in the state $\rho _2$. The first state is prepared a fraction $\eta _1$ of the time and the second state is prepared a fraction $\eta _2 = 1 - \eta _1$ of the time. The ensemble is described by the density matrix

2 $$\begin{aligned} \rho = \eta _1 \rho _1 + \eta _2 \rho _2 \end{aligned}$$

Alice then randomly draws a quantum system from this ensemble and sends it over to Bob.

A more elaborate but equivalent way of describing state preparation is as follows. Alice initially prepares an ensemble of two-qubit states $\rho _{ab} = \eta _1|0\rangle _a\langle 0| \otimes \rho _{1,b} + \eta _2|1\rangle _a\langle 1|\otimes \rho _{2,b}$ and sends particle *b* over to Bob. Then she performs a measurement in the computational basis on the particle in her possession. If she finds the result $|0\rangle$ she knows that Bob’s particle is in the state $\rho _1$ and if she finds the result $|1\rangle$ she knows that Bob’s particle is in the state $\rho _2$. If this is repeated a large number of times, Bob will receive the state $\rho _1$ a fraction $\eta _1$ of the time and the state $\rho _2$ a fraction $\eta _2$ of the time, on average.

Either way, Bob has no knowledge of the actual state he received, all he knows are the prior probabilities, $\eta _1$ and $\eta _2 = 1-\eta _1$. The initial information uncertainty is given by

3 $$\begin{aligned} S_i = - \sum _{j=1}^{2} \eta _j \log \eta _j, \end{aligned}$$

and the initial information is

4 $$\begin{aligned} I_i = 1 - S_i = 1 + \sum _{j=1}^{2} \eta _j \log \eta _j \end{aligned}$$

The question we are addressing here is: How much information can Bob gain by performing measurement(s) on the system he received? To this end we will consider the following general model of quantum measurement. In accordance with the results described in the Introduction, we assume that Bob has *N* detectors described by the set of rank-1 operators $\{\Pi _m|m=1,\ldots ,N\}$ with $d \le N \le d^2$ adding up to the identity operator,

5 $$\begin{aligned} \sum _{m=1}^{N} \Pi _m = {\mathbbm {1}} \end{aligned}$$

The latter condition ensures that given the measured system in any state, one of the detectors will click. The conditional probability that $\Pi _{m}$ clicks, given a system in the state $\rho _{j}$ is calculated using Born’s rule:

6 $$\begin{aligned} P(m|j) = \textrm{Tr}(\rho _j\Pi _m) \end{aligned}$$

In order to have positive probabilities we have to require the positivity (non-negativity) of the detection operators,

7 $$\begin{aligned} \Pi _m \ge 0 \end{aligned}$$

Equations (5) and (7) define a Positive Operator Valued Measure (POVM), which is simply the decomposition of the identity in terms of positive operators, called the elements of the POVM.

Next, we use Bayesian updating, employing Bayes’ theorem, $P(m|j)P(j) = P(j|m) P(m)$, for conditional probabilities. In particular, we apply this formula for the situation when $j=\rho _j$ and $m=\Pi _m$. Then $P(j) = \eta _j$ is the prior probability of state *j*, *P*(*m*|*j*) is the detection probability given in Eq. (6), i.e., detector *m* records an event if state *j* is given, and $P(m) = \eta _1 \textrm{Tr}(\rho _1\Pi _m) + \eta _2 \textrm{Tr}(\rho _2\Pi _m)$ is the total probability that detector *m* records an event. Using Eq. (2), the last expression can be written as

8 $$\begin{aligned} P(m) = \textrm{Tr}(\Pi _m\rho ) \end{aligned}$$

Thus we find that the conditional probability $P(\rho _{j}|\Pi _{m})$, the probability that if detector *m* records an event it is due to the state *j*, can be written as

9 $$\begin{aligned} P(\rho _{j}|\Pi _{m}) \equiv C_{jm} = \frac{\eta _{j} \textrm{Tr}(\rho _{j}\Pi _{m})}{\textrm{Tr}(\Pi _{m}\rho )} \end{aligned}$$

$C_{jm}$ is the confidence (or confidence probability) which is the central quantity in the MC state discrimination strategy.

Equipped with the confidences we next give the information uncertainty for the case when detector *m* clicks. Clearly, using $C_{2m} = 1 - C_{1m}$,

10 $$\begin{aligned} S_{m} = - C_{1m} \log {C_{1m}} - (1-C_{1m}) \log {(1-C_{1m})} \end{aligned}$$

The average uncertainty is

11 $$\begin{aligned} S_{f} = \sum _{m=1}^{N}P(m) S_{m}, \end{aligned}$$

where *P*(*m*) is given by Eq. (8). The information after the measurement is given by $I_{f} = 1 - S_{f}$.

Finally, the information gain from the measurement can be given as

12 $$\begin{aligned} \Delta I = I_{f} - I_{i} = S_{i} - S_{f}, \end{aligned}$$

where $S_{i}$ is given by Eq. (3) and $S_{f}$ is given by (11). Substituting the explicit expressions for the various quantities obtained so far into Eq. (12) it can be shown that this equation is identical to Eq. (1). In this formulation the contributions from the prior and posterior information appear clearly separated. Furthermore, $S_{i}$ is constant for a given set of priors, so it is independent of the measurement we perform. Therefore, optimizing the information gain is equivalent to finding the POVM that minimizes the second term, the information uncertainty $S_{f}$, Eq. (11). As noted before, the information gain that is maximized over all possible measurements is also called the accessible information. In the next section we develop a fully analytical theory that provides the accessible information (optimal solution) in parametric form for all values of the parameters.

### Accessible information for qubits

To treat the optimization problem effectively, we employ the formalism developed for the maximum confidence strategy in Ref.^13^. Equations (11) and (12) are expressed in terms of the confidence probabilities, so they provide a convenient starting point. The method yields the optimal solution analytically in parametric form, in terms of a single parameter.

As the first step we introduce transformed density and measurement operators by the definitions:

13 $$\begin{aligned} \bar{\rho }_j= & {} \eta _j \rho ^{-1/2}\rho _j \rho ^{-1/2}, \end{aligned}$$

14 $$\begin{aligned} \bar{\Pi }_m= & {} \rho ^{1/2} \Pi _m \rho ^{1/2}, \end{aligned}$$

and

15 $$\begin{aligned} \bar{\bar{\Pi }}_m = \frac{\bar{\Pi }_m}{\textrm{Tr}(\bar{\Pi }_m)}, \end{aligned}$$

where $\rho$ is defined in Eq. (2). The transformed states satisfy

16 $$\begin{aligned} {\bar{\rho }}_1 + \bar{\rho }_2 = {\mathbbm {1}} \end{aligned}$$

It follows from this expression that the transformed states ${\bar{\rho }}_{1,2}$ have the same set of eigenvectors. Using the transformed operators we can write the confidence $C_{jm}$, Eq. (9), in the more compact form,

17 $$\begin{aligned} C_{jm} = {\textrm{Tr}}({\bar{\bar{\Pi }}}_m{\bar{\rho }}_{j}) \end{aligned}$$

We wish to maximize the information gain (12) [or the final uncertainty $S_{f}$ (11)] due to the measurement. $S_{f}$ is already in terms of the confidences while the outcome probability becomes

18 $$\begin{aligned} P(m) = {\textrm{Tr}}({\bar{\Pi }}_m) \end{aligned}$$

in terms of the transformed operators. Using Eqs. (14) and (15) it is easy to show from Eq. (5) that the transformed measurement operators ${\bar{\bar{\Pi }}}_m$ satisfy the equation:

19 $$\begin{aligned} \sum _m P(m) {\bar{\bar{\Pi }}}_m = \rho \end{aligned}$$

We note that the transformation $\Pi _m \rightarrow {\bar{\bar{\Pi }}}_m$ is rank preserving. Thus they become rank 1 projectors, not necessarily orthogonal. All we can say is that, as seen from Eq. (19), they correspond to a pure state decomposition of $\rho$, not necessarily in terms of orthogonal pure states. Furthermore, a pair of qubit states are always unitarily equivalent to a pair of real states, their Bloch vectors can be chosen to span the *x*, *z* plane of the Bloch sphere. Therefore, we can assume that ${\bar{\bar{\Pi }}}_m$ is also real. The general expression of a real rank 1 matrix can be written as

20 $$\begin{aligned} {\bar{\bar{\Pi }}}_m=\begin{pmatrix} \cos ^2 \alpha _m &{} \cos \alpha _m \sin \alpha _m \\ \sin \alpha _m \cos \alpha _m &{} \sin ^2 \alpha _m \end{pmatrix} \end{aligned}$$

For the calculation that follows it is convenient to use the common eigenvectors of $\bar{\rho }_1$ and $\bar{\rho }_2$ as basis. Let the eigenvectors be $|1\rangle$ and $|2\rangle$, and the eigenvalues of $\bar{\rho }_1$ be $\lambda _1$ and $\lambda _2$. Eq. (16) immediately gives that the eigenvalues of $\bar{\rho }_2$ are $1-\lambda _1$ and $1-\lambda _2$. Substituting (20) first into (17), we obtain

21 $$\begin{aligned} \cos ^2 \alpha _{m}= & {} \frac{C_{1m}-\lambda _2}{\lambda _1 - \lambda _2} \end{aligned}$$

Without loss of generality we can assume $\lambda _{1} \ge \lambda _{2}$ from where $\lambda _{1} \ge C_{1m} \ge \lambda _{2}$ and $1 - \lambda _{2} \ge C_{2m} \ge 1 - \lambda _{1}$ follow.

Substituting Eq. (20) next into Eq. (19), we find

22 $$\begin{aligned} \sum _{m} P(m)\cos ^2 \alpha _{m}= & {} \rho _{11}, \end{aligned}$$

23 $$\begin{aligned} \sum _{m} P(m)\sin ^2 \alpha _{m}= & {} \rho _{22}, \end{aligned}$$

24 $$\begin{aligned} \sum _{m} P(m)\cos \alpha _{m} \sin \alpha _{m}= & {} \rho _{12} \end{aligned}$$

Here, $\rho _{ij}$ are the matrix elements of $\rho$ in the basis formed by the eigenstates of $\bar{\rho }_1$.

Up to this point our consideration is general as we have not imposed any restriction on the number *N* of POVM elements. However, it has been proven that for a pair of qubit states the optimal measurement is projective^44,46^, that is, $N=2$. Then $\Pi _m = P_m$, where $\{P_m|m=1,2\}$ are rank 1 orthogonal projectors, $P_m P_{m^{\prime }} = P_{m} \delta _{m m^{\prime }}$ ($m, m^{\prime }=1,2$). Therefore, from now on, we deal with the case of two orthogonal detectors and use the notation

25 $$\begin{aligned}{} & {} P(j=1|m=1) \equiv C_{1}, \end{aligned}$$

26 $$\begin{aligned}{} & {} P(j=2|m=2) \equiv C_{2}, \end{aligned}$$

since in this case we want to identify a click in detector $m=j$ with $\rho _{j}$. Hence $C_{j}$ is the probability of “good” events for the corresponding detector.

Using this notation in (21) and then the resulting expression in (22) and (23), some lengthy but straightforward algebra yields

27 $$\begin{aligned} P(1) = \frac{C_2 - \eta _{2}}{C_1 + C_2 - 1}, \ \ \ \ P(2) = \frac{C_1 - \eta _{1}}{C_1 + C_2 - 1} \end{aligned}$$

Substituting (27) into Eq. (11) and then using the resulting expression in (12) we obtain

28 $$\begin{aligned} \Delta I= & {} \frac{C_2 - \eta _{2}}{C_1 + C_2 - 1} [C_1\log (C_1)+(1-C_1)\log (1-C_1)] + \frac{C_1 - \eta _{1}}{C_1 + C_2 - 1} [C_2\log (C_2)+(1-C_2)\log (1-C_2)] \nonumber \\{} & {} -\eta _{1}\log (\eta _{1}) - \eta _{2} \log (\eta _{2}), \end{aligned}$$

which is one of our central results. It expresses the information gain $\Delta I$ entirely in terms of the confidences $C_1$ and $C_2$, and the prior probabilities $\eta _1$ and $\eta _2$. Remarkably, this expression is independent of the structure of the states to be discriminated.

**Figure 1:**
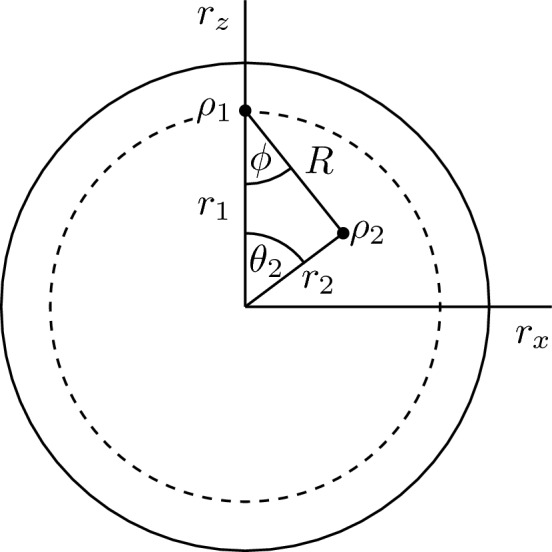
Parametrization of real states: $r_1$ and $r_2$ are the Bloch radii measured from the origin, $\theta _2$ is the polar angle relative to the *z* axis ($\theta _1=0$). *R* is the Euclidean distance of the states $\rho _1$ and $\rho _2$, and $\phi$ is the angle between $r_{1}$ and *R*.

Equations (22) and (23) together with Eq. (21) allowed us to express the information gain entirely in terms of the confidence probabilities. The remaining Eq. (24) represents the main constraint under which (28) should be optimized. From Eqs. (24) and (27) it is easy to obtain the relation

29 $$\begin{aligned} \frac{\sin \alpha _{1} \cos \alpha _{1} - \rho _{12}}{C_{1} - \eta _{1} } + \frac{\sin \alpha _{2} \cos \alpha _{2} - \rho _{12}}{C_{2} - \eta _{2}} = 0 \end{aligned}$$

The first term on the left-hand side is a function of quantities related to state 1 alone, while the second term is a function of quantities related to state 2 alone. Therefore, they separately must be equal to a universal function of the parameters, which we denote by *a*. The function *a* still depends on the parameters of the problem but not on $C_{1}$ and $C_{2}$ and, in order to satisfy (29), it must be antisymmetric under the exchange $1 \leftrightarrow 2$. In terms of *a* we can write

30 $$\begin{aligned} \frac{\sin \alpha _{1} \cos \alpha _{1} - \rho _{12}}{C_{1} - \eta _{1}} = a, \ \ \ \frac{\sin \alpha _{2} \cos \alpha _{2} - \rho _{12}}{C_{2} - \eta _{2}} = -a \end{aligned}$$

This provides a straightforward analytical solution to the entire problem. Substituting $\alpha _{1}$ and $\alpha _{2}$ from Eqs. (21), we obtain the constraint in parametric form. It expresses $C_{1}$ and $C_{2}$ and hence the information gain in terms of the single parameter *a*.

More importantly, however, it leads to a visual geometric solution which is the central result of this paper. It can serve as guide to find the exact solution for any values of the parameters specifying the problem. We introduce the geometric approach in the next subsection and illustrate its power on several examples.

**Figure 2:**
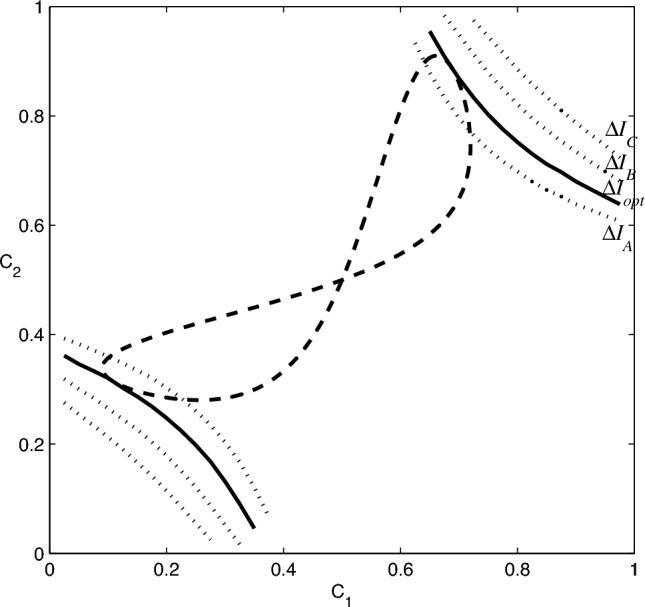
The constraint Eq. (30) (dashed line) and the information gain Eq. (28) (dotted lines) plotted together in the $C_{1},C_{2}$ plane, for various fixed suboptimal values of the information gain ($\Delta I_{A}=0.18$, $\Delta I_{B}=0.3$, $\Delta I_{C}=0.37$). The solid line corresponds to the maximal information gain, $\Delta I_{\max }=0.23129$. Optimal values of $C_{1}$ and $C_{2}$ are the coordinates of the point where the solid line is tangent to the dashed curve (note that there are two sets of optimal solutions). The values of the parameters are $\eta _{1}=\eta _{2}=1/2$, $r_{1}=0.9$, $\theta _{1}=0$, and $r_{2}=0.5$, $\theta _{2}=\pi /4$.

### Geometric optimization

First, we introduce a convenient parametrization of the problem. Recall that two qubit states are always unitarily equivalent to two real states: the corresponding two Bloch vectors span a plane in the Bloch sphere, and this plane can always be unitarily rotated to the $x-z$ plane. We can thus restrict our discussion to this plane, also termed as the Bloch disk. With a further rotation, the Bloch vector of one of the states, say $\rho _1$ can be aligned with the *z* axis. So we assume, without loss of generality, that our states are real from the beginning and the Bloch vector $r_1$ of the first state is along the *z* axis, that is, we use the following parametrization of the states:

31 $$\begin{aligned} \rho _{i} (r_i,\theta _i)=\frac{1}{2} ({\mathbbm {1}}+r_i\sin (\theta _i) \sigma _x + r_i\cos (\theta _i) \sigma _z),\qquad i=1,2. \end{aligned}$$

Here, ${\mathbbm {1}}$ is the two-dimensional identity operator, $\sigma _x$ and $\sigma _z$ are Pauli matrices, $r_i$ is the Bloch radius, and $\theta _i$ is the polar angle of state $\rho _i$, measured from the *z* axis. The parameters are shown in Fig. 1 where $0<r_1,r_2\le 1$, $\theta _1=0$, $0\le \theta _2\le \pi$. In the following, we use these parameters to present our results.

For given fixed values of the parameters, that is, the prior probabilities $\eta _1$ and $\eta _2$ and the eigenvalues of the transformed states, $\lambda _1$ and $\lambda _2$, the constraint (29) (or its parametric version , (30)) can be easily plotted in the $C_1$-$C_2$ plane. This gives us a unique 8-shaped curve on which we have to find the optimal values of $C_1$ and $C_2$. To this end, we notice that the information gain expression, (28), for a fixed value of $\Delta I$ is also a curve in the same plane. If we choose the fixed value $\Delta I$ too large, the two curves do not intersect. Lowering the value of $\Delta I$, for a certain threshold value the two curves become tangent. This value is the maximal information gain $\Delta I_{\max }$ available by the measurement, that is, the accessible information. The procedure is illustrated in Fig. 2. We should also mention that the values $\Delta I < \Delta I_{\max }$ correspond to feasible (suboptimal) measurements, all the way to $C_1=C_2=0.5$, which corresponds to pure guessing.

**Figure 3:**
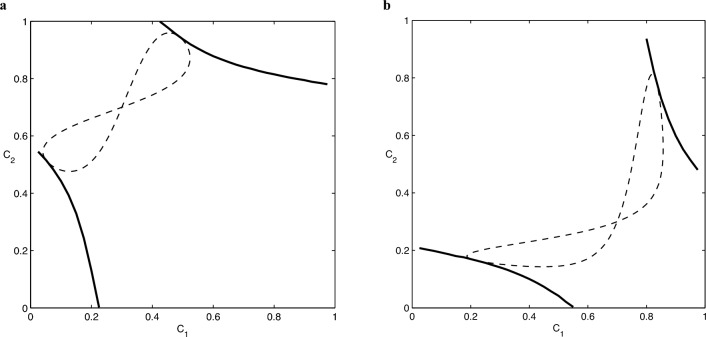
The constraint Eq. (30) (dashed line) and the information gain Eq. (28) (solid line) plotted together in the $C_{1},C_{2}$ plane. The optimal values of the confidences at the upper points of tangency of the curves are (**a**) $C_{1}=0.4879$ and $C_{2}=0.9469$, (**b**) $C_{1}=0.7867$ and $C_{2}=0.8338$. The corresponding maximal information gains are (**a**) $\Delta I_{\max } = 0.2111$ and (**b**) $\Delta I_{\max } = 0.1842$. The parameters of the states are (**a**) $\eta _{1}=0.3$, $\eta _{2}=0.7$, and (**b**) $\eta _{1}=0.7$, $\eta _{2}=0.3$. Other parameters for both figures are $r_{1}=0.9$, $\theta _{1}=0$, and $r_{2}=0.5$, $\theta _{2}=\pi /4$.

Figure 3 shows two examples for the geometric optimization, that is, the constraint (30) and the information gain (28) plotted together in the $C_{1},C_{2}$ plane, for two sets of parameters of the input states. The figure shows that increasing the prior probability $\eta _1$ of the state $\rho _1$ increases the optimal confidence probability $C_1$ of the state while reducing the confidence $C_2$ of the other state.

We find numerically that the optimal values are $C_{1}=0.4879$ and $C_{2}=0.9469$ for the left panel, while $C_{1}=0.7867$ and $C_{2}=0.8338$ for the right panel. It is interesting to note that in the first case both detectors identify $\rho _{2}$ with larger confidence.

In order to interpret these results, we point out that Figs. 2 and 3 are symmetric under reflection about the $C_{1}+C_{2}=1$ line. This property follows from the fact that the information gain, Eq. (28), and the constraint, Eq. (29), are invariant under the substitution $C_{1} \leftrightarrow 1-C_{2}$. In particular, the constraint which is represented by the 8-shaped dashed line in these plots has this symmetry and the point where it intersects itself has coordinates $C_{1}=\eta _{1}$ and $C_{2}=\eta _{2}$. These values correspond to pure guessing with no actual measurement performed and using them in Eq. (28) leads to $\Delta I_{\min }=0$. As noted before, Eq. (28) also gives a relation between $C_{1}$ and $C_{2}$ for a fixed value of the information gain $\Delta I$. When plotted in the $C_{1}-C_{2}$ plane, it exhibits two disjoint segments that are related by the reflection symmetry about the $C_{1}+C_{2}=1$ line. The optimal measurement corresponds to the points where the solid line, Eq. (28), is tangent to the dashed line, Eq. (30). It can be seen that there are two sets of solutions, related by the same symmetry. Feasible measurements are in the region bounded by the two solid lines, yielding a value $\Delta I$ in the range $\Delta I_{\max }> \Delta I > \Delta I_{\min }=0$ as we approach, from either of the boundaries, the point $C_{1}=\eta _{1}$ and $C_{2}=\eta _{2}$ where $\Delta I_{\min }=0$. It should also be noted that, as a consequence of Eq. (21), knowledge of $\lambda _1$ and $\lambda _2$, the eigenvalues of the transformed states in Eq. (16), is sufficient to find the optimum measurement.

In summary, there are two key points of the geometric approach to optimization. First, the constraint (30), linking $C_{1}$ and $C_{2}$, restricts us to a curve in the $C_1 - C_2$ plane, and the maximum of the information gain has to be found along this curve. Second, for a fixed value of $\Delta I$ (such that $0< \Delta I <1$), the expression for the information gain, Eq. (28), also corresponds to a curve in the same plane. If we choose $\Delta I$ too large the two curves may not have any common points. For intermediate values the two curves may have more than one common point. The maximal value $\Delta I_{\max }$ of the information gain is the one for which the two curves become tangent. Geometrically, it corresponds to the unique value of $\Delta I$ for which the $\Delta I(C_{1},C_{2})$ curve becomes tangent to the constraint. This can still happen for more than one point and the coordinates of these points, $C_{1}$ and $C_{2}$, all correspond to optimal measurements, however, the value of $\Delta I_{\max }$ is unique. The actual value can be found numerically. Then we substitute the $C_i$ values corresponding to the tangent points back to (25) and (26) and, using Eqs. (20), (15) and (14), we arrive at the POVM(s) which yield(s) the maximum information about the system (MI POVM).

### Comparison of the MI and ME strategies

Although initially it has been assumed that the minimum error and the maximum information measurements coincide, a careful numerical study revealed that, in general, they are different^49^. Therefore, in the following we present a systematic study of how these two measurements compare. Anticipating the results, we find that the ME and MI measurements coincide for the case of two pure states and, generally, for the case when the two states have the same Bloch radius ($r_1=r_2$) or they are on the same diagonal of the Bloch disk.

In the ME strategy one is looking for measurement operators $\Pi _i$ that maximize the expression

32 $$\begin{aligned} P_S = \eta _1 \textrm{Tr}(\rho _1 \Pi _1) + \eta _2 \textrm{Tr}(\rho _2 \Pi _2). \end{aligned}$$

$P_S$ is the average probability of correctly guessing the input state, aided with the measurement. Introducing

33 $$\begin{aligned} \Lambda = \eta _1 \rho _1 - \eta _2 \rho _2, \end{aligned}$$

and using $\Pi _1+\Pi _2 = \widehat{\mathbbm {1}}$, we can write Eq. (32) in a more compact form,

34 $$\begin{aligned} P_S =\eta _2 + \textrm{Tr}(\Pi _1 \Lambda ). \end{aligned}$$

This expression is clearly maximal if $\Pi _1$ is the projector to the subspace of $\Lambda$ belonging to positive eigenvalues. Hence, $\Pi _2$ will be the projector to the subspace belonging to negative eigenvalues.

**Figure 4:**
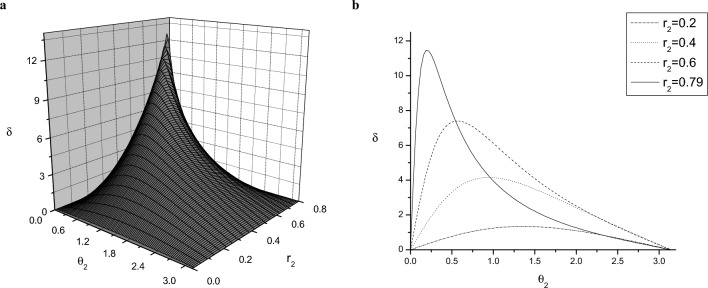
The difference $\delta$ between the ME and MI measurement strategies for the case of two mixed states as a function of (**a**) the Bloch radius $r_2$ and the polar angle $\theta _2$ characterizing the state $\rho _2$, and (**b**) the polar angle $\theta _2$, for representative values of $r_{2}$. $\rho _1$ is a fixed mixed state at $r_1=0.8$, $\theta _1=0$. The difference $\delta$ is measured in degrees, while the polar angle $\theta _2$ is measured in radians.

In order to show the relationship between the ME and MI measurements, we first write Eq. (32) in the form

35 $$\begin{aligned} P_S = P_1 C_1 + P_2 C_2, \end{aligned}$$

which can be obtained if we divide and multiply the first term on the right-hand side of Eq. (32) by $P_{1}$ and the second by $P_{2}$ and use the definition Eq. (8) for the click probabilities and Eq. (9) for the confidences. It has been observed earlier^28^ that the minimum error measurement is also the one that maximizes the average confidence. Furthermore, the relation (27) between the click probabilities and the confidences still holds, so finally, we can write $P_{S}$ as

36 $$\begin{aligned} P_S = \frac{C_2 - \eta _{2}}{C_1 + C_2 - 1} C_1 + \frac{C_1 - \eta _{1}}{C_1 + C_2 - 1} C_2 \end{aligned}$$

This is the cost function to be maximized in the ME measurement. When we compare this to the cost function, Eq. (28), of the MI measurement, we see that there are similarities, e.g., the click probabilities are the same and, in addition, the constraint (29) is also the same for both measurements. Apart from these similarities, the two cost functions are rather different. So, there is no *a priori* reason for the ME and MI measurements to be the same. We will show that they are generally different indeed except for special cases when the input state has some intrinsic symmetry.

What we know is that they are both projective measurements in the *xz* plane. After determining their respective orientations, the difference between the ME and MI strategies can be characterized by the angle $\delta$ between their projectors

37 $$\begin{aligned} \delta = \arccos \sqrt{\textrm{Tr}\Pi _1^{\textrm{ME}} \Pi _1^{\textrm{MI}}}. \end{aligned}$$

Next, we study the dependence of $\delta$ on the structure of the input states. The MI and ME POVMs are determined by using the methods presented previously. Without loss of generality, we will choose the parameters of $\rho _1$ as $r_1$ and $\theta _1=0$ and study the dependence of $\delta$ on the parameters of $\rho _2$, that is, on the polar angle $\theta _2$ and the Bloch radius $r_{2}$ in the Bloch disk, introduced in Fig. 1. In this paper, we focus on the case of equal priors, $\eta _1=\eta _2=1/2$; the case of arbitrary priors will be addressed in a separate publication. Note that, in the following figures, $\delta$ is measured in degrees, while the polar angles are measured in radians.

**Figure 5:**
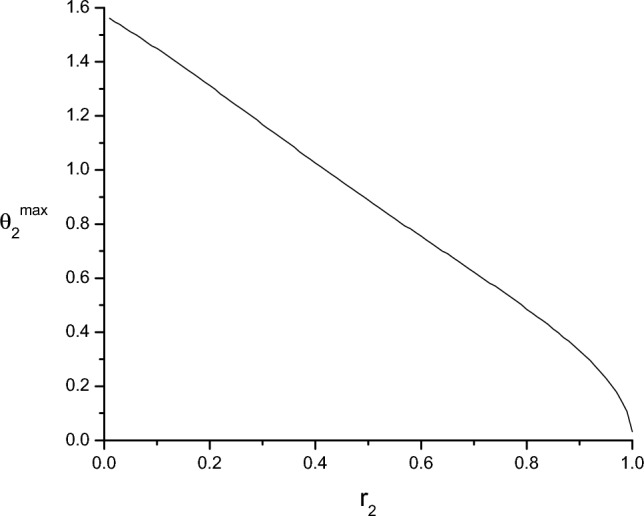
The polar angle $\theta _2^{\max }$ for which the difference $\delta$ between the ME and MI strategies is maximal, as a function of the Bloch radius $r_{2}$ for pure state $\rho _1=\left| 0 \right\rangle \left\langle 0 \right|$ ($r_1=1$, $\theta _1=0$). The polar angle $\theta _2^{\max }$ is measured in radians.

**Figure 6:**
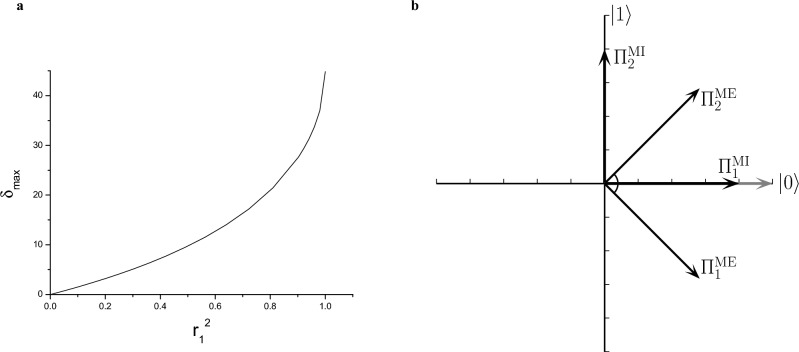
(**a**) The maximum difference $\delta _{\max }$ between the ME and MI strategies as a function of the purity $r_1^2$ of $\rho _1$. The state $\rho _2$ is always close to $\rho _1$ ($r_1-r_2=0.001$). (**b**) The POVMs of the MI (thick vectors) and ME (thin vectors) strategies for two states which are very close to each other on the Bloch disk ($\rho _1=|0\rangle \langle 0|$, $r_2=0.999$, $\theta _2=0.001$).

We consider the general case of two mixed states. Without loss of generality, we assume that $r_2 \le r_1$. Figure 4a shows the difference $\delta$ between the two measurement strategies as a function of the Bloch radius $r_2$ and the polar angle $\theta _2$ characterizing the state $\rho _2$. Figure 4b shows the same quantity as a function of the polar angle $\theta _2$, for representative values of the Bloch radius $r_{2}$. In these figures $\rho _1$ is a fixed mixed state with $r_1=0.8$, $\theta _1=0$. From these figures one can deduce that the two strategies coincide (that is, $\delta =0$) only in the case when the two mixed states have the same Bloch radius ($r_1=r_2$) or they are along the same diagonal of the Bloch disk ($\theta _2=0,\pi$). These rules are valid for any value of $r_1$. Accordingly, the ME and MI strategies coincide for two pure states ($r_1=r_2=1$). For a given $r_{2}$, $\delta$ exhibits a maximum, $\delta _{\max }$, for a certain value of the polar angle $\theta _{2}=\theta _{2}^{\max }$. Figure 5 displays the polar angle $\theta _{2}^{\max }$, corresponding to the maximum difference $\delta _{\max }(r_2)$ between the two strategies, as a function of the Bloch radius $r_{2}$, for the pure state $\rho _1=\left| 0 \right\rangle \left\langle 0 \right|$ ($r_1=1$, $\theta _1=0$). In this figure, the value of the polar angle $\theta _2^{\max }$ decreases linearly, except for values of $r_2$ close to those of the other Bloch radius $r_1$. Note that the value $r_2=1$ for which the two strategies coincide is excluded from the domain of the function $\theta _2^{\max }(r_2)$. Figure 4b shows that by increasing the Bloch radius $r_2$ toward the other radius $r_1$ the value of the maximal difference $\delta _{\max }(r_2)$ between the ME and MI detection strategies also increases. We have found that the function $\delta _{\max }(r_2)$ practically reaches its saturated value $\delta _{\max }$ within a precision 0.01 when $r_1-r_2\lesssim 0.001$. Figure 6a presents the maximum difference $\delta _{\max }$ between the ME and MI strategies as a function of the purity $r_1^2$ of the state $\rho _1$. The state $\rho _2$ is always close to $\rho _1$ ($r_1-r_2=0.001$), that is, $R\rightarrow 0$ in Fig. 1. The figure shows that increasing the purity $r_1^2$ of state $\rho _1$, $\delta _{\max }$ grows nearly exponentially, reaching its maximum when $\rho _1$ is pure. Figure 6b shows the POVMs for this case in the computational basis. The ME POVM is aligned symmetrically around the states. For the MI strategy, however, one of the POVM elements virtually coincides with the pure state, and the other one is perpendicular to rule it out. Although the information provided by the measurements vanishes when the mixed state $\rho _2$ is in the close vicinity of the pure state $\rho _1$, we have found that the MI POVM brings more than twice as much information as the ME POVM for the presented case.

## Discussion

We have developed an analytic method, supplemented by a geometric approach to optimization, for finding the measurement that yields the maximum information gain about a qubit system that is prepared in one of two known states with given prior probabilities. We have determined the parameters of the POVM of the maximum information gain measurement for two arbitrary (pure or mixed) states, prepared with equal prior probabilities, building on previous results that the optimal measurement is always a standard von Neumann measurement for this case. We have compared the maximum information measurement to the minimum error one, and showed that the POVMs of the two measurement strategies coincide exactly only when both states have the same Bloch radius or they are along the same diagonal of the Bloch disk. The case of general priors will be addressed in a subsequent publication.

## Data Availability

The datasets used and/or analyzed during the current study are available from the corresponding author on reasonable request.
